# Assessment of Regional Human Health Risks from Lead Contamination in Yunnan Province, Southwestern China

**DOI:** 10.1371/journal.pone.0119562

**Published:** 2015-04-20

**Authors:** Lu Lu, Hongguang Cheng, Xuelian Liu, Jing Xie, Qian Li, Tan Zhou

**Affiliations:** 1 School of Environment, Beijing Normal University, Beijing 100875, China; 2 Shandong Institute of Standardization, Standardization Technical Research Centre, Jinan city, Shandong province, China; 3 Spatial Science Laboratory, Texas A&M University, College Station, Texas, United States of America; Kaohsiung Chang Gung Memorial Hospital, TAIWAN

## Abstract

Identification and management the 'critical risk areas' where hotspot lead exposures are a potential risk to human health, become a major focus of public health efforts in China. But the knowledge of health risk assessment of lead pollution at regional and national scales is still limited in China. In this paper, under the guidance of 'sources-pathways-receptors' framework, regional human health risk assessment model for lead contamination was developed to calculate the population health risk in Yunnan province. And the cluster and AHP (analytic hierarchy process) analysis was taken to classify and calculate regional health risk and the decomposition of the regional health risk in the greatest health risk region, respectively. The results showed that Yunnan province can be divided into three areas. The highest health risk levels, located in northeastern Yunnan, including Kunming, Qujing, Zhaotong region. In those regions, lead is present at high levels in air, food, water and soil, and high population density which pose a high potential population risk to the public. The current study also reveals that most regional health risk was derived from the child receptors (age above 3 years) 4.3 times than the child receptors (age under 3years), and ingestion of lead-contaminated rice was found to be the most significant contributor to the health risk (accounting for more than 49 % health risk of total). This study can provide a framework for regional risk assessment in China and highlighted some indicators and uncertainties.

## Introduction

With the rapid growth of social economy, huge amounts of lead were discharged into the environment all the time, which had greatly threatened human health, especially for children, in China [1–4]. By now, a growing literature exists trying to assess the health impacts of lead pollution and analyzing the relationship between the lead pollution and health, which have become a major focus of public health efforts in China. Scholars in China conducted some research on the human health risk assessment for some typical lead contaminated sites [5–7] and specific groups of people [8,9]. The assessment of the health risks of a single site may lead to bias, because the results are presented in a non-spatial manner but environmental contaminant distributions vary both spatially and temporally [10]. Therefore regional health risk assessment is required.

To assess regional health risk required the spatial distribution information on the sources, transport, transformation and fate of the contaminants, routes of entry to the body and frequency of contact. Multimedia exposure models provided a useful tool to establish a link among sources, pathways, and receptors data, which offered a useful tool for quantitative environmental health risk assessment at regional and national scales. For example, Niisoe, et al. (2011) used the environment ecological model (Environmental ecological modeling, EEM) to evaluate the health risk of lead exposure in East Asia (including four countries: Japan, Korea, China, Vietnam) [11]. Caudeville, et al. (2012) developed a stochastic spatial multimedia exposure model to assess population exposure at a regional scale in France [12]. However, the researches of regional environmental health risk from heavy metal pollution assessment are rarely seen in China, to date. Only one available presented a risk assessment of atmospheric arsenic contamination in Taiwan Province. In that paper, based on the results of population average daily intake (Average Daily Intake, ADI), the health risk assessment of carcinogenic was mapped [13]. In the context of those assessments, all those studies used the indicator (blood lead levels (BLLs) or hazard quotient (HQ)) to conduct the risk assessment. Although those indicators could effectively reflect the individual potential health risk, the direct use of the indicators was not appropriate for assessing the environmental risk of a contaminant over a large continuous area. For example, such places might exist that where the calculated blood lead levels were high, but it located in a remote area in which few people lived. This might result in bias for the management health risk, especially at regional or national scales. In an attempt to reduce this problem, the regional health risk assessment should be conducted at the population level, and other indicators which could reflect the aggregate population level should be considered.

The present study focused on assessing and zoning health risk caused by lead emission at regional scale, using an integrated assessment approach. The aims of this study were as follows: (1) To evaluate the potential health impacts of lead on the general population in Yunnan province, (2) To provide a better understanding of each exposure pathway, (3) To map the regional health risk and rank the results to identify the 'critical risk areas'. This study can offer research means and approach to quantitatively assess regional human health risk and help local governments to prioritize pollution control and provide support for management of further health risk.

## Materials and Methods

### Ethics Statement

This study was approved by the review board of Beijing Normal University. The samples of drinking water were all collected in residential households and the field studies did not involve any endangered or protected species. The specific locations of sampling sites could be found in supported information (Figure F in S1 File). All residents of the sampling households were informed about the objectives and methods of the study before sampling the drinking water. The legal residents (heads of households) gave verbal consent for permitting our sampling.

In this study, due to the difficulty in sampling the BLLs of the children, the data of children’s BLLs were all calculated by the Integrated Exposure Uptake Biokinetic (IEUBK) model, and the data of BLLs to verify the model were all collected from literatures. Therefore, this research did not involve any children participants, and the statement of the informed consent of children participants did not be addressed.

### The framework of the study

**Fig 1:**
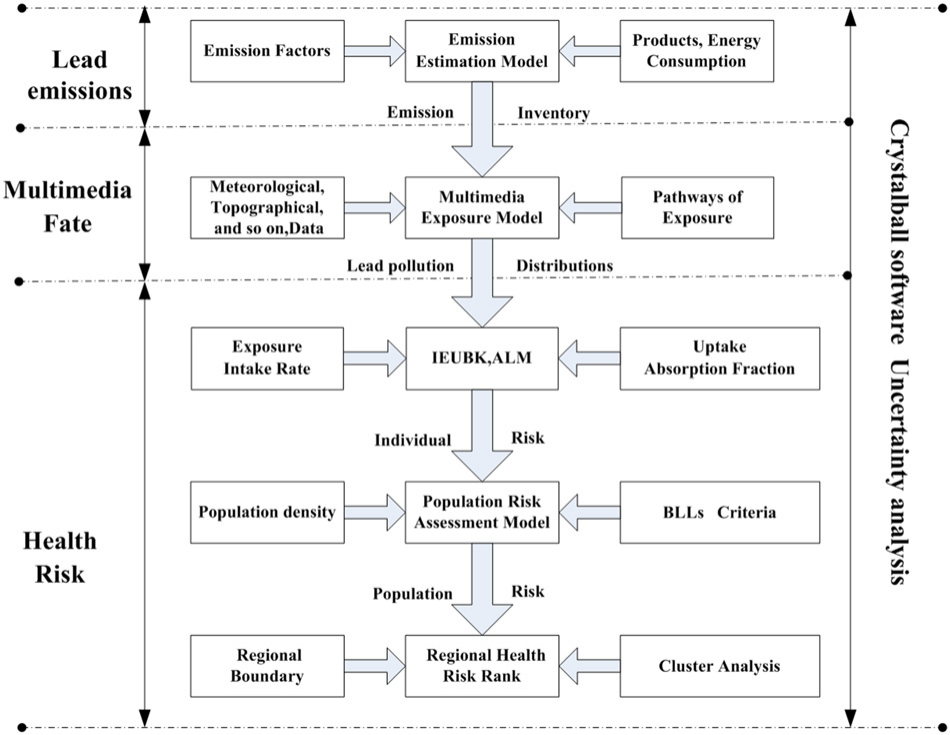
The framework of regional human health risk assessment model for lead contamination.

In this paper, under the guidance of 'sources-pathways-receptors' framework (Fig 1), regional human health risk assessment model for lead contamination in Yunnan province was developed. The regional human health risk assessment model consists of three main sub-models applied in cascade: an emission assessment model (the risk pressure of sources), a multimedia distribution models including atmospheric dispersion model, soil and food Cumulative model (the variability of pathways), and a blood lead levels model (the vulnerability of receptor). ArcGIS 9.3 and SPSS 11 were used to generate process and analyze spatial data from the study area.

Our integrated assessment includes five steps: (1) define the study region (2) develop lead emission inventory of anthropogenic activities in the study area, (3) simulate the lead concentration distribution in multiple environment media model, (4) estimate lead intake and the blood lead levels (BLLs) for individual, (5) estimate population health risk and risk zoning of study region. The following describes steps in detail.

### Definition of Study Region

This study selected Yunnan, province in southern China (31°42’~39°35’ N and 105°29’~111°15’ E, Figure A in S1 File), as the case study. Yunnan is referred to ''the Kingdom non-ferrous metals'', abounds in mineral resources. The production of copper (refined), lead and zinc was 298.4×10^3^(t), 318.7×10^3^(t) and 775.3×10^3^(t) respectively in 2009. In recent years, due to energy consumption, non-ferrous mining exploitation and smelting activities, heavy metal pollution environmental risks to health was increasing, as evidenced by frequent reporting on environmental risks in the local news media, including: lead-zinc poisoning event in Kaiyuan city (2007), blood lead event in Kunming city (2009) and Dali city (2010) that had raised alarms [4].

The regular grid has been generated to assess regional health risk, which had been used widely in dealing with regional health risk caused by complex pollution from multi-sources, multi-pathways and multi-objects [14]. The research domain was a approximate 8.39×10^5^ km^2^ area (900×932 km) centered on the center in Yunnan province (25°37’ N, 101°75’ E), regularly gridded in 5850 cells (75×78 cells, 12km cell size).

### Lead Emission Inventory

To compute the lead emission from anthropogenic activities in Yunnan province, the emission estimation model was employed. According to the emission estimation model, annual lead emission in Yunnan is estimated on the basis of economic statistics for 2009, as fuel consumption and industrial production multiplied by corresponding emission factors.

$$E_{ij}=Q_{ij}\times F_{ij}$$ (1)

Where: E is the amount of lead emissions; Q is the production of fuel consumption and industrial production; F is the emission factor; i is the region (municipality or autonomous region); and j is the emission source classified by economic sector.

Based on the methods of emission factors, the emission estimation model divided the emission sources into six categories, including: coal combustion, motor vehicle gasoline combustion, iron and steel production, cement production, non-ferrous metal manufacturing and other sources. More detailed discussion on model evaluation issues (such as the formula) can be noted in literature [15]. The result of emission assessment was presented in (Figure B and Figure C in S1 File).

### Multimedia Fate

Humans are vulnerable, in general, to a multitude of lead in air, food, soil and water, so the exposure pathways considered in this assessment include inhalation and the ingestion of soil, food and water. The information on the transport, transformation and fate of lead in those media were required to assess the health risk. Because the infrastructure and the monitoring network of lead concentration in different media at regional scale in China are still under development, multimedia distribution models including atmospheric dispersion model, soil and food cumulative model were used to estimate environmental compartment and exposure media concentrations.

#### Lead concentration in air

The PSU/NCAR mesoscale model (known as MM5) and CALPUFF models were integrated to predict the ambient lead concentration and the deposition in Yunnan in 2009. The MM5 model was used to provide 4D meteorological data required by the CALPUFF model. The details of the required data for MM5 (i.e., topography data, 3D first-guess meteorological fields and the meteorological measurements, as well as the physical options used were the same as those described in Cheng et al. 2012 [16]. In the horizontal, three-level nested-grid architecture was used for the implementation of MM5-CALPUFF. Modeling domain 1 and domain 2 were used for the MM5 simulation, with spatial resolutions of 108km ×108km and 36km ×36km, respectively. Domain 3 was used for the MM5 and CALPUFF simulation and covered the area of interest (i.e., Yunnan with a spatial resolution of 12km ×12km). The grid cells for the three domains were 27km×31km, 40km×40km and 79km×76km, respectively. Vertically, 35 sigma levels were used for the MM5 simulation. The top height was 35km at the pressure of 100 mbar. These 35 levels were condensed into 11 layers using the CALMET processor to match the format required by the CALPUFF. Land use and elevation data came from the US 'Geological Surveys EROS Data Center' database. In addition, the lead emission inventory for the CALPUFF simulation is calculated in section 2.2.

#### Lead Concentrations in soil

The lead concentration in soil was composed of the background lead concentration in soil (B) and the cumulative lead concentration in soil (C_SC_). The cumulative lead concentration in soil due to the total deposition D_tot_ resulting from the contributions of both the dry (D_dry_) and the wet (D_wet_) deposition flux, as well as to the loss from soil through processes such as soil erosion, degradation, etc, was calculated by the following equation [17], and probability distributions for these parameters derived from the literature data are reported in (Table A in S1 File). Moreover, detailed information of the calculated lead concentration in soil could be found in (Figure D and Figure E in S1 File).

$$C_{S}=C_{SC}+B=\frac{D_{tot}[1-\exp(-k_{s}\times T)]}{z\times k_{s}\times BD}+B$$ (2)

#### Lead Concentrations in drinking water

Due to the limited data, the 98 samples of drinking water from the tap and well in the residential area (Figure F in S1 File), two main pathways of drinking water exposure for urban residents and rural residents respectively, were collected at random to represent the entire region in Yunnan Province. And the samples were analyzed by inductively coupled plasma-mass spectrometry (ICP-MS) (Agilent 7500i, Agilent Scientific Technology Ltd., USA). GIS is used to combine the location information with the geographical concentration distributions (Figure F in S1 File).

#### Lead Concentrations in diet

The intake of grains, vegetables etc. is one of the main exposure pathways of exposure to lead pollutants, and the lead pollutants in air, water and soil can be accumulated and enter the body through diet. In this study, four kinds of agricultural products were considered: wheat, rice, corn, vegetables, which were the major food types consumed in Yunnan province. Bioaccumulation models [17] were used to estimate the lead concentration in production through the bio-concentration factors methods and the basic equation as followed. The bio-concentration factor collected from the literature data was presented in (Table B in S1 File).

$$C_{F}(i,j)=BCF(i)\times C_{i}(j)$$ (3)

Where: C_F_ is the concentration of lead in food, mg/kg; BCF is the bio-concentration factor; C_S_ is the the concentration of lead in soil; i is the different type of food; j is the different region.

### Multimedia intake and individual health risk

In this study, the children (age range, from 0 to 7 years) and blood lead levels were selected as an individual health risk assessment objective and indicator, respectively, because of these facts: (1) Children were sensitive to lead contamination, because children behavior, especially their hand—mouth habits, and resistance were significantly different from that in adults; (2) In study area, children are direct the victims group in all lead poisoning events (3) Blood lead levels was the most widely used index of internal lead body burdens associated with potential adverse health effects. However, due to the difficulty in sampling the BLLs of the children, the Integrated Exposure Uptake Biokinetic (IEUBK) model was used to calculate the lead blood levels of children, and data of BLLs from literatures were used to verify the model. So, this research did not involve any children participants.

The IEUBK models were developed by the U.S. Environmental Protection Agency and recommended as risk assessment tools for children due to lead exposure, and have been widely applied to evaluate the lead risk in different countries. The IEUBK model utilizes four interrelated modules (exposure, uptake, biokinetic, and probability distribution) to estimate blood lead levels in children exposed to lead contaminated media. Intake by ingestion or inhalation is expressed in ug Pb/day and is calculated by multiplying the lead media concentration value (Pb_air_, Pb_soil_, Pb_food_, Pb_water_) with the media Intake Rate (IR) value (breathing rate or ingestion rate expressed as volume of media per day). Absorption is calculated by multiplying the lead intake value with the absorption fraction (AF) value (expressed in % of lead intake). And the biokinetic component can simulate the absorption, transportation, metabolism and elimination in the body, and estimates BLLs finally [18,19].

Data on physiological parameters used for the calculation of lead intake and absorption are reported in (Table C and D in S1 File). A value of 37% has been used for lead absorption via lungs after intake via inhalation, and the AF values of 45% and 30% have been used for lead absorption via ingestion for children of age < 1and 1< age < 7 years, respectively. These values have been chosen following the recommendations of Leggett and World Health Organization (WHO) [20,21]. And the pharmacokinetic parameters in the biokinetic component were cited from literature [9]. In this paper, the author suggested that the body weight and the other four key parameters were set as a truncated normal distribution.

### Population and Regional Health Risk

#### Population Health Risk

To understand the risk of lead exposure on public health at regional scales, it is necessary to conduct the regional health risk assessment at the population level. Population risk shall refer to the probability distribution of the actual health impact that may be generated by the imposition of a given set of individual risks. The number of individual and inter-individual variability, which could cause uncertainty of the assessment results [22], should be considered as indicators.

In this study, the number of children at different ages (i.e., 1 to 2 years) was selected as an indicator to calculate the population health risk in each cell. Due to the limit of data, the individual variability at each age did not take into account in this study. It is generally accepted that the higher blood lead level, the higher health risk [21,23]. On the basis of China's national blood lead level diagnostic criteria, the weight value was taken accounted into the assessment of regional health risk (Table E in S1 File). Detailed population data at different ages were collected for each of 16 regions, and assigned to grid cells (12km×12km) within the total region of Yunnan province (Table F in S1 File). The equation of calculating population weighted health risk from lead pollution was as follows:

$$R=\overset{7}{\underset{i=1}{\sum }}BLLs(i)\times P(i)\times W(i)$$ (4)

Where: R is the population health risk in each cell; BLLs is the blood lead level; P is the number of exposure population; W is the weight value; i is the population at the age of i.

#### Regional Health Risk

In China, risk management was implemented mainly in the administrative unit boundaries by local government. Environmental health risk classification could provide useful information for policy and decision-maker to perform contamination control and risk management. In order to calculate the total health risk in each administrative unit, the risk of grid cells in each administrative unit-region was summed by GIS. The cluster analysis was taken through Squared Euclidean distance method to classify the regional health risk.

### Uncertainty analysis

To accommodate the uncertainties associated within the calculation process, Monte Carlo simulation technique was used based on Crystal ball software (Oracle Corporation, Vallejo, US). Crystal Ball used a Monte-Carlo simulation in order to propagate the distributions calculating the risk 10000 times by randomly drawing values from the probability distribution functions of the input data and the model parameters. Probability density functions were adopted for different parameters from the literature data and summarized in (Table A, B,C and D in S1 File).

## Results and Discussions

### Heavy metal levels in different environmental media

The results of calculated lead concentration in multimedia (air, soil and diet) were confirmed by the comparisons between the simulated and measured data (Table F and G in S1 File; Figure H and I in S1 File). The average fractional difference |f| values of Pb in air and soil were less than 0.33, indicating generally good agreement with actual levels of Pb in air and soil. However, there was a slight underestimation of lead concentration in diet.

The estimated annual average lead concentration levels in air ranged from 0.02 μg/m^3^ to 0.56 μg/m^3^, and the lead concentration in air in most of grid cells were under the national of atmospheric secondary standard limit value 0.50 μg/m^3^, except those grid cells where high lead emission came from (i.e., Qujing, Kunming, Honghe region). The highest grid cells (with lead concentration 0.56μg/m^3^) located in Kunming region which was much higher than those of Chengdu (0.38μg/m^3^), Tianjing (0.27μg/m^3^) and Beijing (0.080μg/m^3^) (Figure J in S1 File).

The results of prediction of lead levels in soil showed the seriously polluted grid cells (>300mg/kg) located in high lead deposition areas (such as Kunming region) and high background areas (such as Dali region) (Figure K in S1 File). And the trends of lead concentration in food were agreed with the concentration in soil, for the linear relationship between lead concentration in food and soil was used. Lead contamination levels changed depending on different types of food (Table H in S1 File). The rice contained higher lead concentrations ranged from 0.086mg/kg to 1.88mg/kg and most exceeding the national limit (0.3mg/kg). The SD (standard deviation) showed higher varieties compared with other foods (ranged from 0.352 to 0.983 in different regions). Different from the rice, the estimated concentration in wheat, corn, vegetables rarely exceeded the national limit 0.2mg/kg. Because rice was the main source of food source in study areas, the lead concentration in rice should be raised alarm.

The concentrations of lead in drinking water from the analyzed results of 98 samples showed that most of samples (94 samples) were under the detection limit (0.1μg /L), and the concentrations in the remaining 4 samples ranged from 14 to 43 μg/L (Figure H in S1 File) and all samples come from the well water in the mining area. The results suggested the drinking water in most of Yunnan was safe, as the lead concentration was far less than the national drinking water quality criteria (10μg/L), but the drinking water in the mining area should be raised alarm, for the drink water from the well was polluted and the lead concentration was 1.4 to 4 times more than the national criteria.

### The BLLs of the child receptor

**Fig 2:**
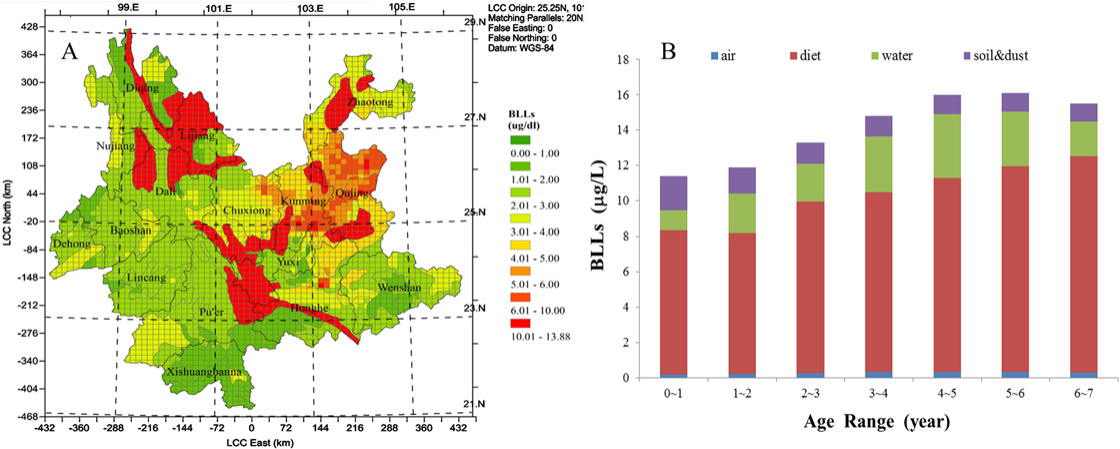
A: Spatial distribution of the estimated BLLs for the child receptor; B The contribution of BLLs from different exposure pathways according to age in the highest grid cell.

In this study, BLLs was taken as an indicator for individual health risk assessment, and was performed for each cell in the calculation domain. The BLLs map plotted using GIS tool in Fig 2A, which displayed the spatial distribution of the geometric mean value of the BLLs of children (age ranged 0 to 84 month). Meanwhile, in Kunming city, the modeled BLLs data of children were compared with the observed data, and two sets of data indicated the estimated BLLs were within a reasonable range (Figure H in S1 File). According to the map (Fig 2A), the value of BLLs ranged from 2.04 to 13.88 μg/dl in the study area. In eastern Yunnan (i.e., southern Kunming, Yuxi, Honghe, and north western Dali, Lijiang region), the BLLs ranged from 6.76 μg/dl to 13.88μg/dl. But the relatively low BLLs (ranged from 2.04 to 4.05 μg/dl) were observed in southern Yunnan province, like Xisuangbanna, Puer region. On the whole, the spatial variation trend was similar to the lead concentration in soil.

The highest BLLs value is estimated in a grid cell located in the north-western part of Qujing region (the values of calculated BLLs with a geometric mean of 13.88 μg/dl, 75.62% above 10μg/L). Due to the different physiology (intake factors, absorption and transfer rates, receptor vulnerability) and habits (sanitation situation, eating habits), the contribution of each pathway to BLLs of children (age range 0~7 year) varied according to age (Fig 2B). The contribution from inhalation (the pathway of air, from 1.78% to 2.18%) is much lower compared with ingestion (the pathways of diet, water, soil, from 97.82% to 98.24%). Among three pathways of ingestion, diet is the most important exposure pathway, and the contribution rate increased from 68.30% to 78.96% with age. The BLLs of children in this cell mainly was controlled by the high concentration of lead in food. High lead concentration in the food was observed mainly due to enrichment from the soil. It should be noted that lacking extensive crop cultivations, it was assumed that the population consumes home-grown corps, and high concentration in food causing from high concentration lead in soil, which led to rather high calculated risk in the study area. And the education and occupation of parents, the residential environment such as house floor, community, and the eating habit lead to the individual variability of BLLs for children. Due to the data limit, those factors were not taken accounted in to this study.

### Population health risk and risk map zoning

**Fig 3:**
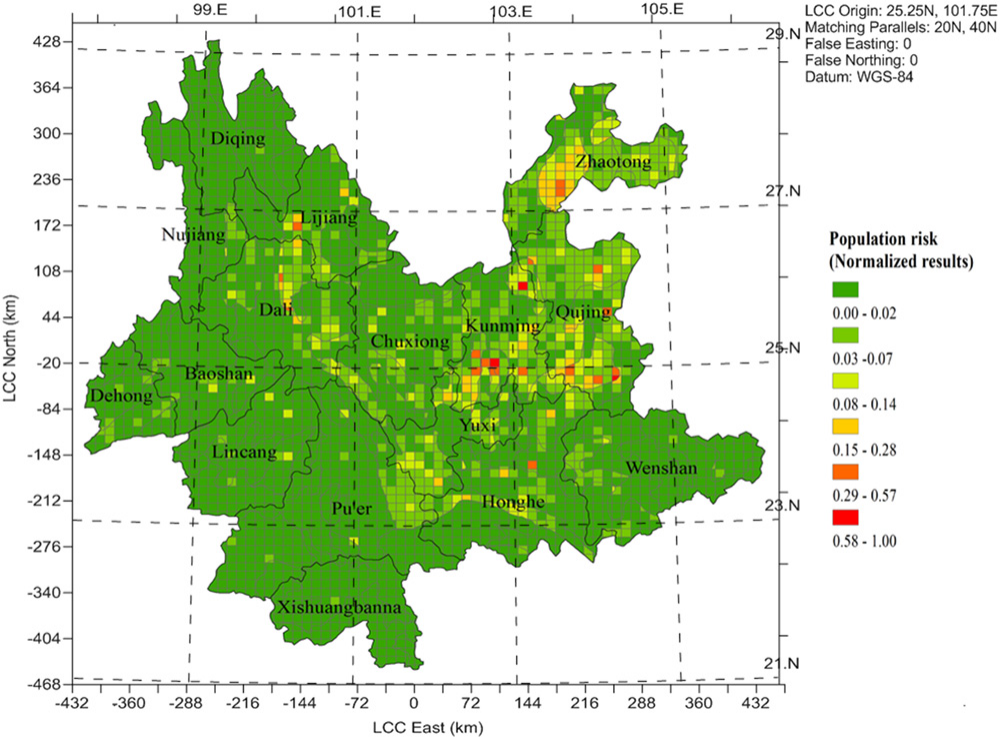
The Spatial distribution of population health risk in Yunnan province.

The spatial distribution of population health risk to lead-related pollution in Yunnan province was presented in Fig 3. The normalization results showed that population and BLLs controlled the distribution of the population health risk. Compared to the BLLs of the children map (Fig 2A), the map (Fig 3) of population health risk changed significantly in Lijiang, Yuxi, Chuxiong regions. Due to high background value of soil (>250mg/kg), the high BLLs (>8μg/dl) was estimated in those regions, but the estimated population health risk was low. Because those areas mostly located in deserted places, although high lead concentration was accumulated in environmental media, little lead could enter in to the human body to endanger human health. Consist with the trend of the BLLs, the relatively high risk cells located in Kunming region, followed by Qujing region and all located in eastern Yunnan (the highest population density and emission). And low population health risk also observed in south and north western Yunnan province, due to the minimal pollution of lead.

**Fig 4:**
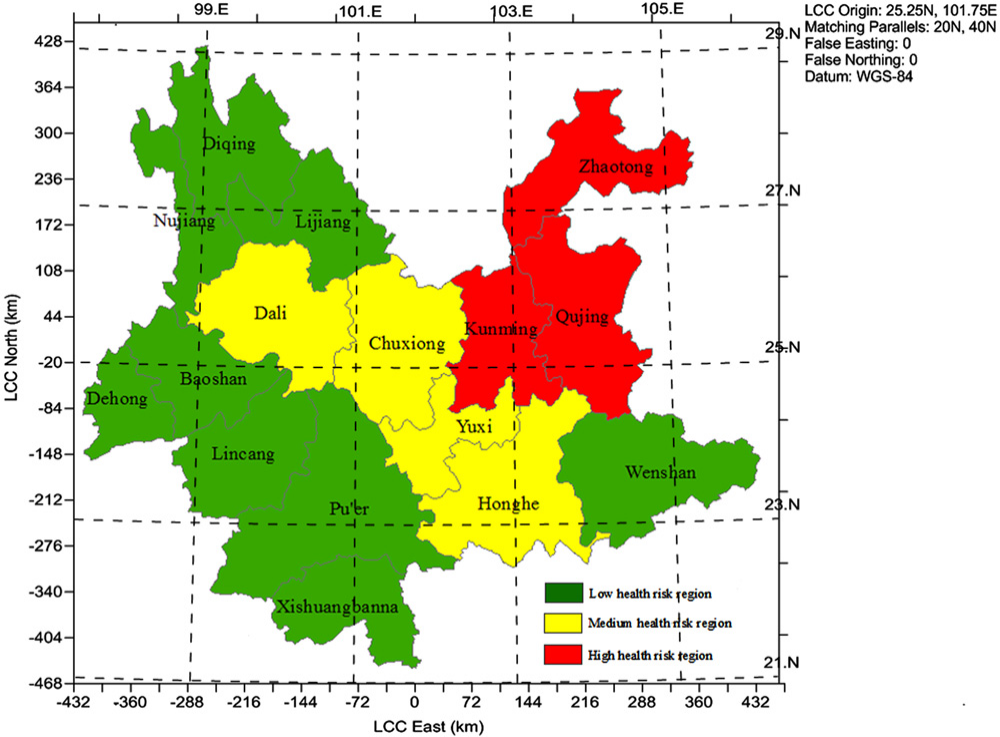
Spatial distribution of the risk classification by regions in Yunnan province.

The regional health risk was calculated by summing the population health risk in each administrative unit-region. The cluster analysis was taken through Squared Euclidean distance method to classify the relative health risk. The result of cluster analysis was shown in Fig 4 and Figure K and L in S1 File. On the whole, the health risk decreased from the eastern Yunnan (Kunming region) to western Yunnan (Diqing region), and from the northeastern (Zhaotong region) to the southern Yunnan (Xishuangbanna region). Yunnan province can be subdivided into three areas. The first area showed relatively slight health risk level and located in southern and north western Yunnan including Xisuangbannan, Dehong, Diqing, Nujiang Wenshan, Puer, Baoshan, Lijiang, Lincang region, accounting for 74.3% of the total land area in Yunnan. Due to relatively few lead emission and low environmental media concentration, children who lived in those regions show relatively low individual risk (BLLs of children ranged from 3.04 μg/dl to 0.46 μg/dl far under the acceptable national level 10μg/L), as well as low population density caused low regional health risk. The second area displayed medium health risk level and most regions located in the centre of Yunnan province adjacent to the first area, including Honghe, Yuxi, Dali, Chuxiong region. The very high soil background concentration was observed in those regions, causing high BLLs (the BLLs more than 8 μg/dl), but low population density resulted in relatively low risk, compared to the third area. The third area was at the high health risk level, located in northeastern Yunnan province, including Kunming, Qujing, Zhaotong region. Those regions were the industrial and economic centers of Yunnan, where more than 67.69% lead of totals was emitted and high soil background concentration also observed which caused high individual risk. These results suggested that great efforts are required to control the lead pollution in eastern Yunnan province (i.e., Kunming, Qujing, Qujing region), and reducing lead emission pollution and concentration in foods was an effective way to control the population risk of lead pollution.

### The relative contribution to the regional health risk

**Fig 5:**
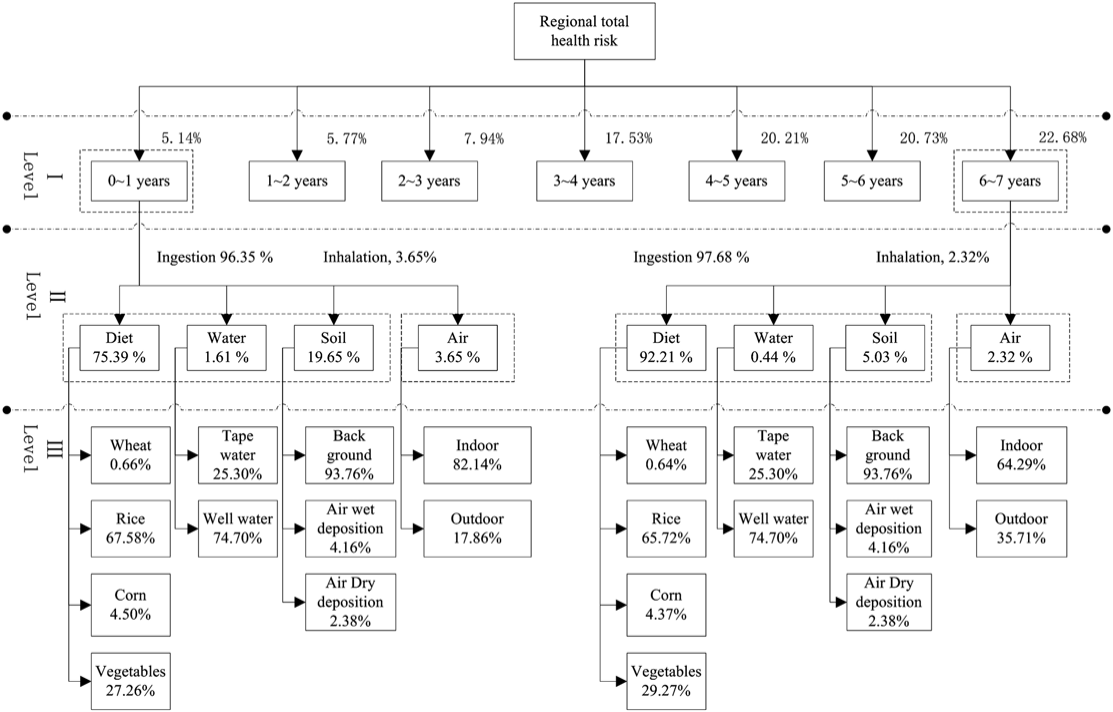
Total risk variance decomposition in Kunming region.

The analytic hierarchy process (AHP) method which has been widely used to analyze environmental impact assessments [24], and evaluate hazardous waste transportation [25], was used to analyze the relative contribution to the regional health risk in Kunming region (the greatest health risk area). Three hierarchy structures was set according to the composition of the regional health risk, and summarized in Fig 5. Level I was the relative contribution of two receptors for the regional health risk; Level II was the relative contribution of exposure pathways for the two receptors; Level III was the relative contribution of the input model parameters within each exposure pathway.

The results of level I showed that the contribution to the total risk altered considerably for child receptors with different ages (i.e., the child receptors (6~7 years) accounted for 22.68% of total risk, more than four times of the receptors (0~1 years). And this indicated that the older children were more vulnerable to the damaging effects of lead than the younger children receptors in this region, due to increasing the time of exposure and the intake of lead.

Results of level II illustrated that contribution from inhalation (the pathway of air) is much lower compared with ingestion (the pathways of diet, water, soil) for the two receptors. Among three pathways of ingestion, diet is the most important exposure pathway, with average contributions from 74.66% (1~2 years) to 92.21% (6~7 years) of ingestion pathway for the child receptors. Owing to the different physiology (intake factors, absorption and transfer rates, receptor vulnerability) and habits (sanitation situation, eating habits), the contribution of each pathway to individual risk varied significantly. For example, the child receptors(6~7 years), the risk variance is almost exclusively related to the diet (92.1%); While the child receptors (0~1 years) diet is still the main contributor to the variance but at a lower extent (75.39%) because of a greater contribution from ingestion of contaminated soil (20.68%).

The results of level III displayed that rice was the main contribution (from 56.01% (1~2 years) to 67.76% (6~7 years) for the child receptors with different ages) to diet exposure pathway, followed by vegetables, with an average contribution altered from 27.02% (3~4 years) to 39.70% (1~2 years). The reasons could attribute to the relatively high bio-concentration factor and consumption of rice in Kunming region. Due to the high lead concentration (ranged from 23.58 to 36.45 μg/L) and the direct source of drinking water for a large number of rural populations, the well water accounted overall for about 74.70% of the water exposure path. For only twenty years air deposition was calculated and the lead concentration of soil in 1990 was used for back ground concentration in this study, 6.54% contribution to soil exposure pathway was obtained for air deposition (wet deposition accounted for 66.72%). Due to lack the information of lead concentration in indoor air, it was assumed that the indoor air concentration for a fraction ranging between 50% and 70% of outdoor concentration, following IEUBK model suggestions. Because the longer time consumed indoor environment (18 ~ 23h, the default value in the IEUBK model), the larger contribution to air exposure pathway was offered from indoor environment.

### Sensitivity and Uncertainty Analysis

Crystal Ball software (adopted Monte Carlo method) was utilized to quantify the varieties and the relative contribution of the different input parameters to the assessment results. The results of those analyses were shown in (Table I and J in S1 File). Sensitivity analysis showed that the dominant contributor to BLLs variance is the variability of BCF of rice (32.58% contribution to variance). The second most significant contribution to the variance is the absorption factor for lead via ingestion (AF-ingestion, 31.2% contribution to variance). Furthermore, background value of soil and soil bulk density contributes to BLLs variance with 22.02and 8.73%, respectively. These results confirmed that the ingestion of rice is most likely to be responsible for any shift in the magnitude and spread of risk distribution.

The overall uncertainties for BLLs of children are estimated at -70% to 136%. The pathways of diets and water are identified as the main contributor of uncertainties in BLLs calculation (-75% to 159%, -41% to 197%) among all of the four pathways. It can be mainly attributed to limited information on bio-concentration factors and high variability of lead concentration in water (0.1 to 43 μg/l, respectively). And accumulation of uncertainties from soil (-25% to 100%) also accounted for large uncertainty of the assessment results. There have been few studies conducted on the real key parameters (such as, bio-concentration factors used in multimedia distribution models, and the absorption factor for lead via ingestion used in IEUBK models) for the study region, to date. In this study, those factors are mainly compiled by the average values of measurements from foreign and domestic studies on the other regions. It is inevitable that using those factors to conduct the risk assessment will contain high uncertainties (i.e., -67% to 179% for rice). And the results of sensitivity analysis show that the parameters are under a greater impact on the results of the risk assessment (i.e., BCF of rice accounting for 32.58% variance of the BLLs calculation). Therefore, long-term field testing and continuous monitoring to get the key parameters used for health risk assessment in Yunnan province, warrant further investigation.

Uncertainties in environmental media (i.e., soil, air, water) used partly arise by the use of grid cell values. Averaging data on a certain spatial resolution, such as the grid cell (12km × 12km) used in this study, will neglect the variation in soil properties within each cell unit and therefore will neglect possible the extreme concentration for some parts of a grid cell unit (i.e., compared with the observed data, the lower concentration in air from simulated result was found). Ideally, both the range and the uncertainty of the data used in each calculated unit (the grid cell) should be taken into account. However, there are very few intensive tracer field experiments and literatures available in the study region for evaluating the uncertainties of the simulated results. Obviously, region-wide monitoring network of lead distribution in the environment and human health risk should be established and improved, because those works are the key element in developing new policies on reduction of lead contaminants.

## Conclusion

In this paper, under the guidance of 'sources-pathways-receptors' framework, regional human health risk assessment model for lead contamination in Yunnan province was developed. According to the results of the assessment, Yunnan province can be divided into three areas. The high health risk level, located in northeastern Yunnan province, including Kunming, Qujing, Zhaotong region. In those regions, lead is present at high levels in air, food, water and soil, which pose high potential risk to children receptor.

The results show that the most serious pollution grid cell in north-western Qujing region, and the average lead concentration was 0.56μg/m^3^ in air, 319.56mg/kg in soil, 0.32mg/kg in food and 43μg/l in water, respectively, all beyond the national limit. And the estimated BLLs of children receptor (age range 0 to 6 years) in this cell were 11.30 above 10μg/dl (commonly considered as acceptable level in risk assessment practice). And the diet was the most important exposure pathway for children receptors, which accumulated lead from soil. The older children were more vulnerable to the damaging effects of lead than the younger children receptors in this region, due to increasing the time of exposure and the intake of lead. Thus the diet pathways and lead concentration in foods, specifically in those regions where high concentration lead in soil were observed, should be given priority attention for regional health risk management. However, it should also be pointed out that our risk model used conservative assumptions and some key parameters cited from literatures, which generated uncertainties to our risk assessment results. To achieve a more reliable risk assessment in regional, basic researches for the localization of input parameters and region-wide monitoring network of lead distribution in the environment are necessary.

## Supporting Information

S1 File Supporting figures, tables and text. (DOC)
