# Supplementary material for: Assessment of Regional Human Health Risks from Lead Contamination in Yunnan Province, Southwestern China
Source: PLoS One. 2015 Apr 20;10(4):e0119562. doi: 10.1371/journal.pone.0119562 (PMC4404351; doi:10.1371/journal.pone.0119562)
Supplement: S1 File — (DOC) [file pone.0119562.s001.doc]

**Supplementary information**

**Assessment of Regional Human Health Risks from Lead Contamination in Yunnan Province, Southwestern China**

Hongguang Cheng^*1^, Lu Lu^1^, Xuelian Liu^1^, Jing Xie^1^, Qian Li^2^, Tan Zhou^3^

1 School of Environment, Beijing Normal University, Beijing 100875, China

2 Shandong Institute of Standardization, Standardization Technical Research Centre

3 Spatial Science Laboratory, Texas A&M University, College Station, TX, USA

* Corresponding author

**S - Part 1. Estimation of lead emissions in Yunnan province**

In this study, atmospheric emissions of lead from anthropogenic activities were calculated by combining the regional-level statistical data and detailed emission factors, which are specified by six different economic sectors (coal combustion, motor vehicle gasoline combustion, combustion of oil, iron and steel production, cement production, non-ferrous metal manufacturing ). The algorithm of a bottom-up emission inventory can be expressed by the following equation. The result of estimates of past and current emissions of lead in Yunnan province in 2009 is illustrated in (Figure A in S1 File):

where: E is the amount of lead emissions; C is the lead content of the raw coal consumed by each regions in Yunnan province; A is the amount of coal consumption; F is the fraction of lead released from the coal combustion facility; P and P_FGD_ are the lead removal efficiency of dust removal equipment and flue gas desulfurization (FGD) devices, respectively; i is the region (municipality or autonomous region); and j is the emission source classified by economic sector, combustion facility, and use of precipitators and SO_2_ control devices; K_Pb_ is the lead content in gasoline, Q_gasoline_ is the gasoline consumption; 0.76 indicates that 76% of the lead contained in gasoline is emitted into the air; Q_smelting_ is the production of non-ferrous metal, namely, Pb, Zn and Cu for the purposes of the present study; C is the coefficient of pollution production for smelting technology m; and f is the efficiency of PM control facility n; Q_others_ is fuel consumption or industrial production of other sources; F is the emission factor of other sources.

To describe the emissions more accurately in this study, the spatial allocation method was used to allocate regional level emissions into grid cells with a resolution of 12km×12km (Figure C in S1 File). Power plants and large-scale metallurgical enterprises are considered to be point sources, and the associated lead emissions are directly allocated into the grid cells according to their latitude and longitude. Other anthropogenic sources and miscellaneous small-scale plants are treated as area sources. According to the proportion of population and industrial gross domestic product (GDP) by regions, lead emissions from area sources are allocated into the 12km×12km grid cells in each region.

**S - Part 2 Multimedia fate**

**The calculation of lead concentration in soil**

In this study, only the data of the soil lead concentration in 1990 was obtained (Figure E in S1 File). In order to estimate the data of soil concentration in study year (2009), the lead concentration in soil in 1990 was set to the background value, the prediction of the twenty years cumulative lead concentration in topsoil was carried out and shown in Figure E in S1 File, and the estimated soil concentration in study year (2009) was shown in Figure F in S1 File.

**S – Part 3 Models Validation**

In this study, to evaluate the model performance, comparisons with observations were made. Unfortunately, no monitoring network has yet been developed in the study area (Yunnan Province). Therefore, calculated data from models were compared with published observation results.

1. **Comparison between observed and simulated data in multimedia**

The concentration of lead in air was monitored seasonally at two sites in Gejiu City. The information of Pb in soil and diet were collected by previous studies, which were summarized in Table F and Table G in S1 File, respectively. Meanwhile, the fit of modeled values to observed values was evaluated by the fractional difference f and those results were showed in Figure J in S1 File:

$f=\frac{V_{mdl}-V_{obs}}{V_{mdl}+V_{obs}}$ (R1)

Where: V_mdl_ and V_obs_ are the modeled data and observed data, respectively. |f| < 0.33 indicates an error factor less than 2.

According to the result of File S1.Figure S10, the calculated lead concentrations in air and soil generally agreed well with measurements in studied areas as indicated by |f| values less than 0.33 (Figure J in S1 File). Meanwhile, comparison of the modeled and measured spatial distributions of lead concentration in soil in the study area was presented in Figure K in S1 File. In general, their spatial distribution patterns were similar. Non-ferrous metal smelting areas (Lanping, Zutang, Gejiu) were high lead contaminated areas (Figure K in S1 File).

However, there was a slight underestimation of lead concentration in diet (Figure J in S1 File). The possible reasons for this result were as follows: Firstly, the observed data of diet were collected mostly in non-ferrous metal smelting areas. In those areas, the elevated lead concentrations in soil often were underestimated in some sites by the model (Figure K in S1 File). Moreover, in this study, the linear relationship between lead concentration in food and soil was used. However, this relationship was rather simplified which often led to the calculation results contained some uncertainties. Additionally, observations reflect concentrations at a single point location but the modeled estimates reflect the average over a 12 km×12 km grid cells.

1. **Comparison between observed and simulated data in BLLs**

In Kunming city, the estimated results were compared with the observed data which were collected from literatures. Due to the limited observed data, the equation (R1) was not used to evaluate the model performance. Simple comparison was conducted between the calculated and modeled BLLs. The results showed that the calculated BLLs (mean value of 11.83 μg/dl, 60.09% above 10μg/dl, in 2009) was slightly higher than literature data (mean value of 10.88 μg/dl, 46.9% above 10μg/dl, in 2001; 8.39μg/dl, 24.9% above 10μg/dl, in 2003). Considered compared to 2004, lead emissions increased by 36.96% in 2009, and the blood lead events were reported frequently in the local media in recent year, so the estimated BLLs were within a reasonable range. And the results suggested that with the increase of lead emissions, lead pollution may pose a high health risk to children in those regions.

**Tables**

**Table A Probability density functions for the parameters in Eq. (1)**

**Table B Bio-concentration factors of lead from different diets**

**Table C The estimate lead concentration in the food**

**Table D Physiological parameters: intake rates (IR) of food**

**Table E the China's national blood lead level diagnostic criteria and weight value in Eq. 3**

**Table F Physiological parameters: intake rates (IR) of inhalation, water, Soil**

**Table G The observed lead concentration in soil in Yunnan province collected from previous studies**

**Table H The observed lead concentration in food in Yunnan province collected from previous studies**

**Table I Uncertain analysis for key input parameters**

**Table J Sensitivity analysis for BLLs calculation**

**Table A Probability density functions for the parameters in Eq. (1)**

| **Parameters** | **Distribution model and parameters** | | **references** |
| --- | --- | --- | --- |
| **Soil bulk density**  **(BD g/cm^3^)** | Lognormal | M_g_-1.39 |  |
|  |  | SD_g_-1.11 |  |
|  |  | [0.93-1.84] |  |
| **Soil loss constant**  **(ks year^-1^)** | normal | M-0.03 |  |
|  |  | SD-0.01 |  |
| **Soil mixing depth**  **(z cm)** | uniform | 1-5 (urban) |  |
|  |  | 10-20 (rural) |  |

**Table B Bio-concentration factors of lead from different diets**

| **Foods** | **Distribution model and parameters** | **references** |
| --- | --- | --- |
| **rice** | N[-5.08,0.89]* |  |
| **wheat** | N[-7.00,1.07]* |
| **corn** | N[0.003,0.0006] |  |
| **vegetable** | N[0.0016,0.0013] |  |

N = normal [mean, standard deviation]; * normal [mean (natural logarithm (BCF)), standard deviation(natural logarithm (BCF))]

**Table C Physiological parameters: intake rates (IR) of** inhalation, water, Soil

| **Intake rates,age** | **Inhalation(m3/day)** | **Water intake (ml/day)** | **Soil intake (mg/day)** |
| --- | --- | --- | --- |
|  |  |  |
| **0 to <1 years** | LN[5.4,1.71] | TR [324,360,396] | TR [0.1,25,50] |
| **1 to <2 years** | LN[8,1.71] | TR [243.9,271,298.1] | TR [0.1,25,50] |
| **2 to <3 years** | LN[9.5,1.71] | TR [285.3,317,348.7] | TR [0.1,25,50] |
| **3 to <4 years** | LN[10.9,1.71] | TR [342,380,418] | TR [0.1,25,50] |
| **4 to <5 years** | LN[10.9,1.71] | TR [342,380,418] | TR [0.1,25,50] |
| **5 to <6 years** | LN[10.9,1.71] | TR [342,380,418] | TR [0.1,25,50] |
| **6 to <7 years** | LN[10.9,1.71] | TR [342,380,418] | TR [0.1,25,50] |

TR = triangular [min, mode, max], LN = lognormal [geometric mean, geometric standard deviation];the distribution model and deviation options were cited from literature .

**Table D Physiological parameters: intake rates (IR) of food unit: g/day**

| **Intake rates, age** | **wheat** | **rice** | **corn** | **vegetable** |
| --- | --- | --- | --- | --- |
| **0 to <1 years** | LN(2.06,1.5) | LN(31.07,1.4) | LN(4.29,1.5) | LN（48.72,1.5） |
| **1 to <2 years** | LN(2.51,1.5) | LN(37.89,1.5) | LN(5.25,1.5) | LN（104.4,1.5） |
| **2 to <3 years** | LN(5.38,1.5) | LN(81.20,1.5) | LN(11.20,1.5) | LN（140.3,1.5） |
| **3 to <4 years** | LN(6.15,1.5) | LN(92.83,1.5) | LN(12.88,1.5) | LN（144,1.5） |
| **4 to <5 years** | LN(7.52,1.5) | LN(113.59,1.5) | LN(15.66,1.5) | LN（182.7,1.5） |
| **5 to <6 years** | LN(8.05,1.5) | LN(121.47,1.5) | LN(16.75,1.5) | LN（213.89,1.5） |
| **6 to <7 years** | LN(9.42,1.5) | LN(142.01,1.5) | LN(19.61,1.5) | LN（245.86,1.5） |

LN = lognormal [geometric mean, geometric standard deviation]; the diet data came from the Yunnan Statistical Yearbook,2009; the distribution model and and deviation options were cited from literature .

**Table E the China's national blood lead level diagnostic criteria and weight value in Eq. 3**

| BLLs（ug/L） | Status | Weights |
| --- | --- | --- |
| 0～50 | Normal | 1 |
| 50～99 | Normal^*^ | 2 |
| 100～199 | High lead levels | 4 |
| 200～249 | Mild lead poisoning | 8 |
| 250～449 | Moderate lead poisoning | 16 |
| ≥450ug/L | Severe lead poisoning | 32 |

*Recently, the US Centers for Disease Control and Prevention (CDC) recommended that the BLLs for children blow 5μg/dl was relatively safe;

**Table F The observed lead concentration in soil in Yunnan province collected from previous studies**

| **Regions** | **Sites** | **Numbers** | **Year** | **Pb (mg/kg)** | | | **Reference** |
| --- | --- | --- | --- | --- | --- | --- | --- |
|  |  |  |  | (Mean ± SD) | Min | Max |  |
| **Baoshan** | Changning | 16 | 2008 | 40.26 ± 3.44 | - | - |  |
|  | Shidian | 17 | 2008 | 49.57 ± 3.65 | - | - |  |
|  | Tengchong | 22 | 2011* | 52.2 | 30.2 | 109 |  |
|  | Longyang | 22 | 2011* | 29.5 | 11.4 | 61.3 |  |
|  | Changning | 23 | 2011* | 34.6 | 26.1 | 43.9 |  |
|  | Baoshan | 10 | 2009* | 22.44 ± 9.32 | - | - |  |
| **Dali** | Yunlong | 20 | 2010 | 364.5 ± 164.20 | 66 | 528 |  |
|  | Xiaguan | 45 | 2008 | 14.9 | 10.3 | 23 |  |
| **Honghe** | Gejiu | 51 | 2009 | 1185.48±490.72 | 379.63 | 2472.53 |  |
|  | Yuanyang | 35 | 2004 | 43.19 ± 8.87 | 30.04 | 59.31 |  |
|  | Mengzhi | 8 | 2009* | 18.21 ± 16.54 | - | - |  |
| **Kunming** | Dianchi | 45 | 2002~2003 | 56.98 ± 39.62 | 23.67 | 264.79 |  |
|  | Chenggong | 12 | 2009* | 17.02 ± 12.53 | - | - |  |
|  | Guandu | 8 | 2009* | 16.43 ± 5.00 | - | - |  |
| **Lincang** | Yunxian | 13 | 2008 | 65.49 ± 4.12 | - | - |  |
|  | Fengqing | 16 | 2008 | 51.47 ± 2.34 | - | - |  |
| **Nujiang** | Lanping | 18 | 2010 | 742 ± 413.62 | 1260 | 306 |  |
| **Puer** | Jingmai | 16 | 2008 | 30.76 ± 1.56 | - | - |  |
|  | Zutang | 15 | 2008 | 1497 ± 30.66 | - | - |  |
| **Qujing** | Nanpan River | 12 | 2011 | 47.00 ± 44.00 | 183 | 22 |  |
|  | Nanpan River | 12 | 2011 | 26.00 ± 9.32 | 45 | 14.6 |  |
|  | Qujing | 6 | 2009* | 11.83 ± 1.82 | - | - |  |
| **Xisuangbanna** | Yiwu | 18 | 2008 | 38.66 ± 2.25 | - | - |  |
|  | Rendugang | 15 | 2008 | 28.87 ± 1.99 | - | - |  |
|  | S | 14 | 2008 | 39.33 ± 1.24 | - | - |  |
|  | Bulang | 16 | 2008 | 21.64 ± 2.89 | - | - |  |
|  | Bohai | 14 | 2008 | 26.20 ± 1.62 | - | - |  |
| **Yuxi** | Tonghai | 19 | 2009* | 36.46 ± 14.69 | 22 | 68.3 |  |
|  | Yuxi | 8 | 2009* | 31.45 ± 13.88 | - | - |  |

Note: “*” represented the received the manuscript time; “-” represented no data;

**Table G The observed lead concentration in food in Yunnan province collected from previous studies**

| **Region** | **Sites** | **Numbers** | **Year** | **Diet** | **Pb (mg/kg)** | | | **Reference** |
| --- | --- | --- | --- | --- | --- | --- | --- | --- |
|  |  |  |  |  | (Mean ± SD) | Min | Max |  |
| **Kunming** | Kunming | 42 | 2003* | Vegetables | 0.864 | 0.078 | 3.286 |  |
| **Honghe** | Gejiu | 51 | 2009 | Rice | 0.63 ± 0.14 | - | - |  |
|  |  |  | 2009 | Corn | 0.94±0.29 | - | - |  |
|  |  |  | 2009 | vegetables | 0.3±0.14 | - | - |  |
| **Nujiang** | Lanping | 19 | 2008 | Rice | 1.89 | 0.15 | 3.27 |  |
|  |  | 37 | 2008 | Rice | 4.57 | 0.01 | 15.98 |  |

Note: “*” represented the received the manuscript time; “-” represented no data;

**Table H The estimate lead concentration in the food**  Unit：mg/kg

| **Regions** | **Vegetables** | | | | **Wheat** | | | | **Rice** | | | | **Corn** | | | |
| --- | --- | --- | --- | --- | --- | --- | --- | --- | --- | --- | --- | --- | --- | --- | --- | --- |
|  | **Min** | **Max** | **Mean** | **SD** | **Min** | **Max** | **Mean** | **SD** | **Min** | **Max** | **Mean** | **SD** | **Min** | **Max** | **Mean** | **SD** |
| **Baoshan** | 0.078 | 0.14 | 0.102 | 0.021 | 0.062 | 0.112 | 0.082 | 0.017 | 0.267 | 0.482 | 0.352 | 0.072 | 0.093 | 0.168 | 0.123 | 0.025 |
| **Chuxiong** | 0.06 | 0.75 | 0.268 | 0.286 | 0.048 | 0.6 | 0.214 | 0.229 | 0.206 | 1.88 | 0.921 | 0.983 | 0.072 | 0.9 | 0.321 | 0.343 |
| **Dali** | 0.015 | 0.75 | 0.274 | 0.296 | 0.025 | 0.6 | 0.219 | 0.237 | 0.057 | 1.58 | 0.944 | 0.919 | 0.025 | 0.9 | 0.329 | 0.356 |
| **Dehong** | 0.078 | 0.14 | 0.107 | 0.025 | 0.062 | 0.112 | 0.085 | 0.02 | 0.267 | 0.482 | 0.367 | 0.086 | 0.093 | 0.168 | 0.128 | 0.03 |
| **Diqing** | 0.06 | 0.75 | 0.281 | 0.297 | 0.048 | 0.6 | 0.225 | 0.237 | 0.206 | 1.88 | 0.667 | 0.842 | 0.072 | 0.9 | 0.337 | 0.356 |
| **Honghe** | 0.034 | 0.75 | 0.198 | 0.25 | 0.027 | 0.6 | 0.158 | 0.2 | 0.116 | 1.88 | 0.681 | 0.859 | 0.041 | 0.9 | 0.238 | 0.3 |
| **Kunming** | 0.023 | 0.75 | 0.21 | 0.245 | 0.021 | 0.6 | 0.168 | 0.196 | 0.013 | 1.88 | 0.721 | 0.881 | 0.042 | 0.9 | 0.252 | 0.293 |
| **Lijiang** | 0.06 | 0.75 | 0.463 | 0.33 | 0.048 | 0.6 | 0.37 | 0.264 | 0.206 | 1.88 | 0.893 | 0.837 | 0.072 | 0.9 | 0.556 | 0.397 |
| **Lincang** | 0.046 | 0.11 | 0.097 | 0.018 | 0.037 | 0.088 | 0.078 | 0.015 | 0.159 | 0.377 | 0.335 | 0.063 | 0.056 | 0.131 | 0.117 | 0.022 |
| **Nujiang** | 0.078 | 0.75 | 0.214 | 0.224 | 0.062 | 0.6 | 0.171 | 0.179 | 0.267 | 1.88 | 0.736 | 0.77 | 0.093 | 0.9 | 0.257 | 0.269 |
| **Puer** | 0.046 | 0.75 | 0.174 | 0.211 | 0.037 | 0.6 | 0.139 | 0.168 | 0.159 | 1.88 | 0.599 | 0.724 | 0.056 | 0.9 | 0.209 | 0.253 |
| **Qujing** | 0.046 | 0.75 | 0.195 | 0.213 | 0.037 | 0.6 | 0.156 | 0.17 | 0.159 | 1.88 | 0.671 | 0.731 | 0.056 | 0.9 | 0.234 | 0.255 |
| **Wenshan** | 0.034 | 0.14 | 0.08 | 0.039 | 0.027 | 0.112 | 0.064 | 0.031 | 0.116 | 0.482 | 0.274 | 0.134 | 0.041 | 0.168 | 0.096 | 0.047 |
| **Xisuangbannan** | 0.046 | 0.14 | 0.07 | 0.027 | 0.037 | 0.112 | 0.056 | 0.021 | 0.159 | 0.482 | 0.241 | 0.092 | 0.056 | 0.168 | 0.084 | 0.032 |
| **Yuxi** | 0.025 | 0.75 | 0.355 | 0.33 | 0.02 | 0.6 | 0.284 | 0.264 | 0.086 | 1.88 | 0.62 | 0.836 | 0.03 | 0.9 | 0.425 | 0.396 |
| **Zhaotong** | 0.06 | 0.75 | 0.251 | 0.281 | 0.048 | 0.6 | 0.201 | 0.225 | 0.206 | 1.88 | 0.863 | 0.968 | 0.072 | 0.9 | 0.301 | 0.338 |

**Table I Uncertain analysis** for key input parameters

| **Parameters** | **Description** | **Unit** | **Value** |
| --- | --- | --- | --- |
| **C_s_** | concentration of soil | mg/kg | 114.38(-25%,100%) |
| **C_rice_** | concentration of rice | mg/kg | 0.98(-67%,179%) |
| **C_corn_** | concentration of corn | mg/kg | 0.34(-68%,123%) |
| **C_vegetable_** | concentration of vegetable | mg/kg | 0.29(-45%,125%) |
| **C_wheat_** | concentration of wheat | mg/kg | 0.23(-48%,112%) |
| **C_diet_-BLLs** | BLLs for the pathway of diet | μg/dl | 5.74(-75%,159%) |
| **C_air_-BLLs** | BLLs for the pathway of air | μg/dl | 0.61(-65%,38%) |
| **C_soil_-BLLs** | BLLs for the pathway of soil | μg/dl | 0.85(-51%,92%) |
| **C_water_- BLLs** | BLLs for the pathway of water | μg/dl | 0.31(-41%,197%) |
| **BLLs** | BLLs for child receptor | μg/dl | 7.52(-70%,143%) |

**Table J**  Sensitivity analysis for BLLs calculation

| **Assumptions** | **Description** | **Contribution To Variance** | **Sensitivity** |
| --- | --- | --- | --- |
| **BCF- rice** | Bio-concentration factor of rice | 32.58% | 0.5534 |
| **AF-Ingestion** | Absorption factor for lead via ingestion | 31.20% | 0.5415 |
| **B** | Background value of soil | 22.02% | 0.4549 |
| **BD** | Soil bulk density | 8.73% | -0.2864 |
| **Ca** | Concentration of lead in air | 2.57% | 0.1554 |
| **BCF- Vegetable** | Bio-concentration factor of Vegetable | 2.32% | 0.1476 |
| **Ks** | Soil loss constant | 0.17% | -0.0403 |
| **IR-Vegetable** | Intake rate of vegetable | 0.11% | 0.0321 |
| **Z** | Soil mixing depth | 0.07% | -0.0255 |
| **Dtot** | Deposition flux of lead | 0.07% | 0.0253 |
| **IR-Rice** | Intake rate of rice | 0.04% | 0.0201 |
| **IR-air** | Intake rate of air | 0.02% | 0.0147 |
| **IR-water** | Intake rate of water | 0.02% | 0.0132 |
| **IR-corn** | Intake rate of corn | 0.02% | 0.0128 |
| **BCF-wheat** | Bio-concentration factor of wheat | 0.02% | 0.0128 |
| **IR-soil & dust** | Intake rate of soil & dust | 0.02% | -0.0128 |
| **BCF-Corn** | Bio-concentration factor of corn | 0.01% | 0.0104 |
| **AF-inhalation** | Absorption factor for lead via inhalation | 0.01% | 0.0093 |
| **IR-wheat** | Intake rate of wheat | 0.01% | 0.0080 |
| **Cw** | Concentration of lead in water | 0.00% | 0.0037 |

**Figures**

**Figure A Locations of the study areas**

**Figure B Lead emissions by region from different sources in Yunnan province in2009**

**Figure C Spatial distribution of emission intensity of lead in the study area**

**Figure D The background of lead concentration in the soil in 1990 (shaded, mg/kg)**

**Figure E Horizontal distributions of predicted cumulative lead concentration in topsoil from atmospheric deposition of lead (shaded, mg/kg)**

**Figure F The distribution of lead concentration in drinking water from samples**

**Figure G The distribution of population in Yunnan Province in 2009**

**Figure H Scatter plot of lead concentrations in Multimedia (air, soil and diet) for the model results and observations in Yunnan province.**

**Figure I Comparison of the modeled (white-black color contour) and measured (scaled circles) spatial distributions of lead concentration in soil in the study area.**

**Figure J Distribution of the modeled annual mean concentrations of lead in air in the study area, the color scale is in units of μg m^-3^**

**Figure K The concentration of lead in the top soil after third years deposition**

**Figure L Cluster Analysis conclusion of regional health risk level of lead pollution in Yunnan province**


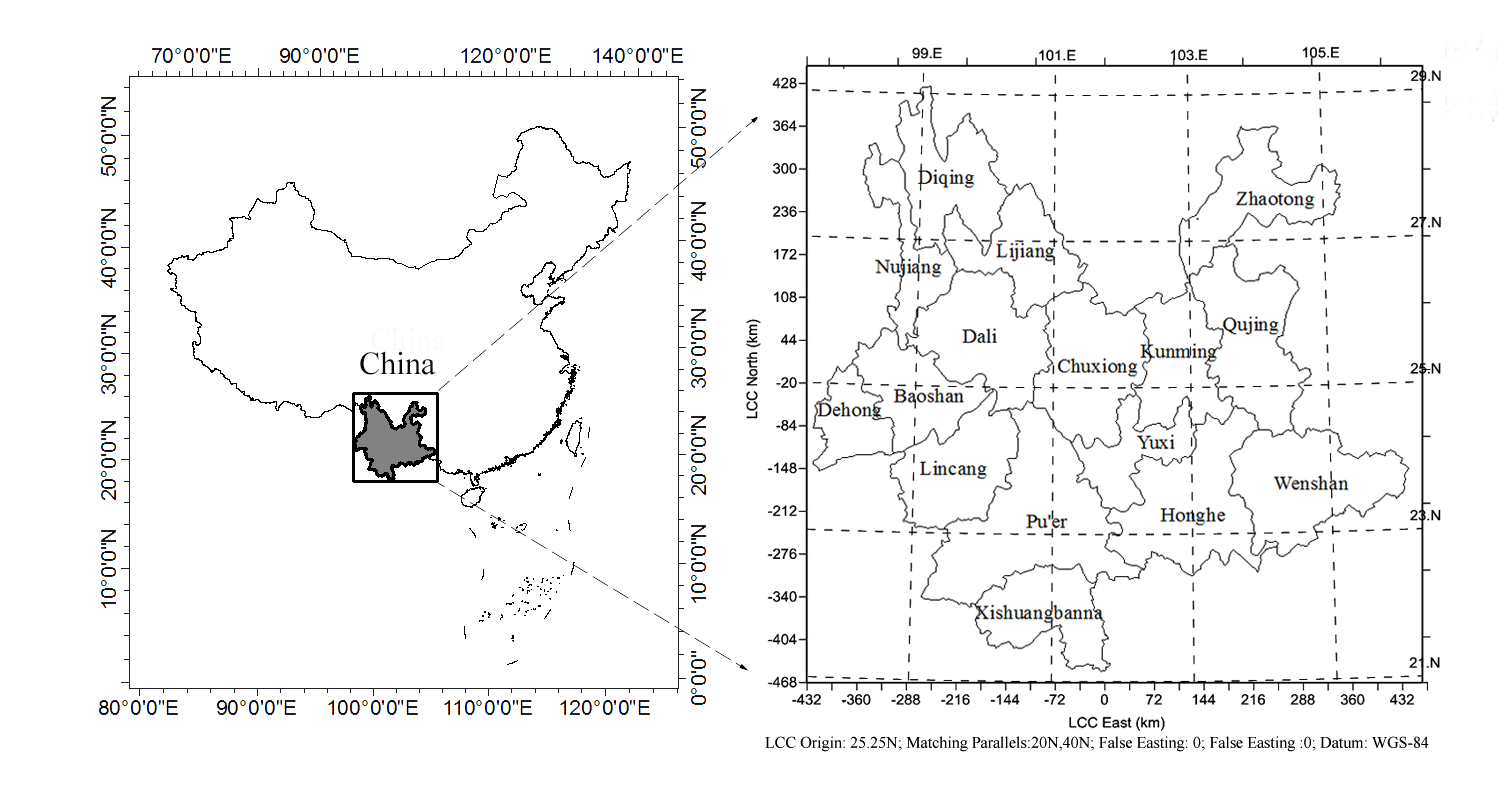


**Figure A Locations of the study**

areas
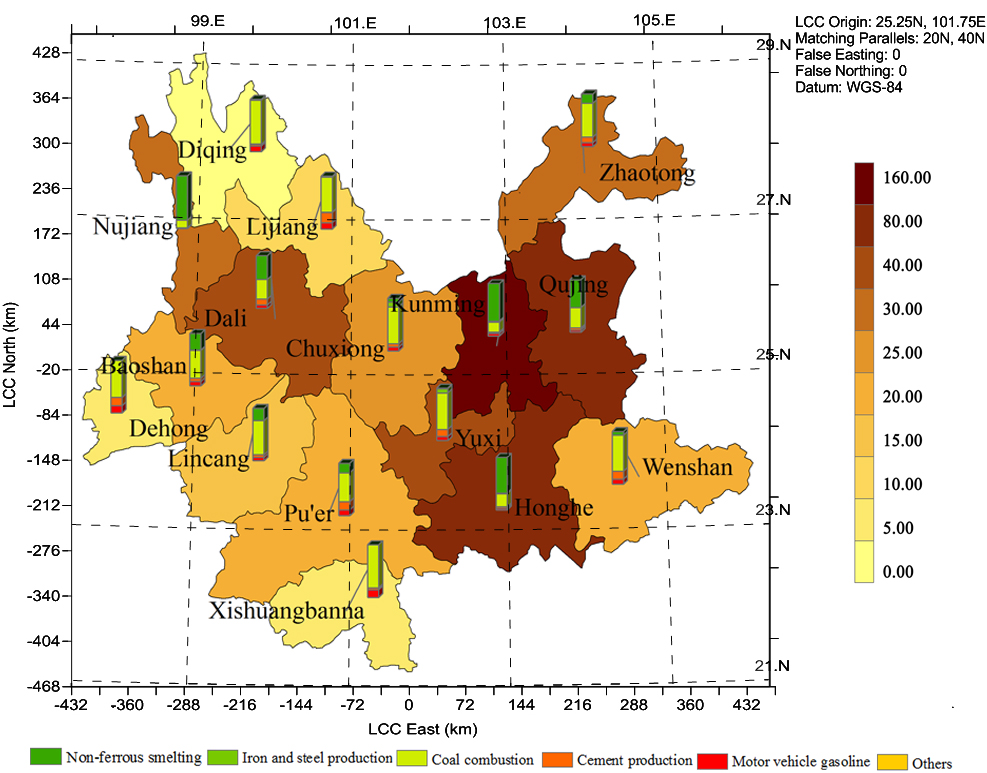


**Figure B Lead emissions by region from different sources in Yunnan province in2009.**


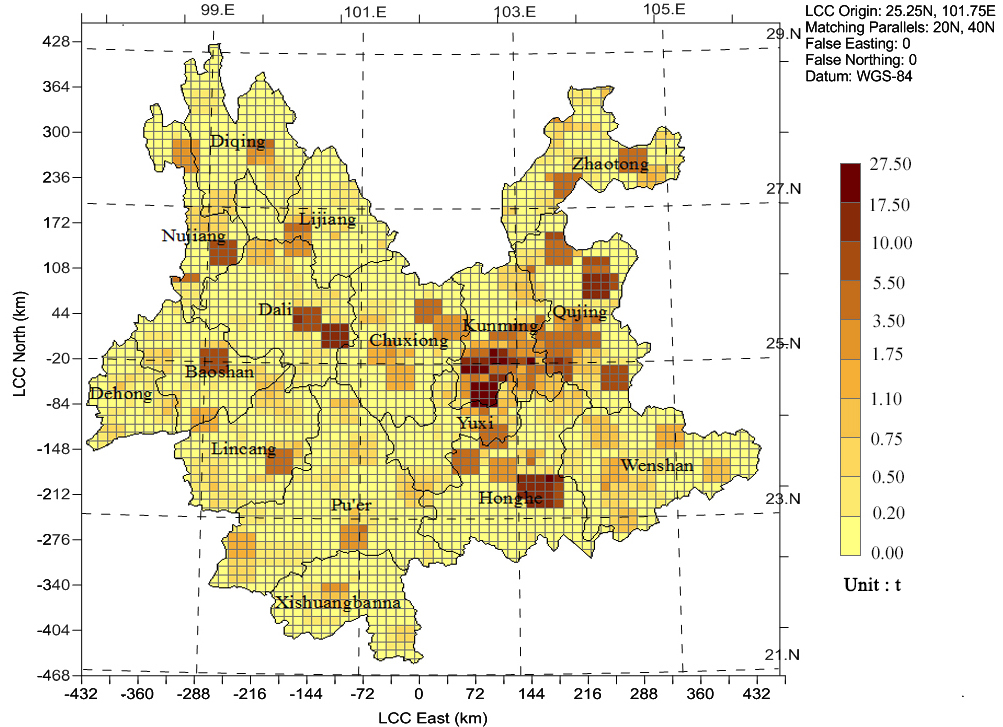


**Figure C Spatial distribution of emission intensity of lead in the study area**


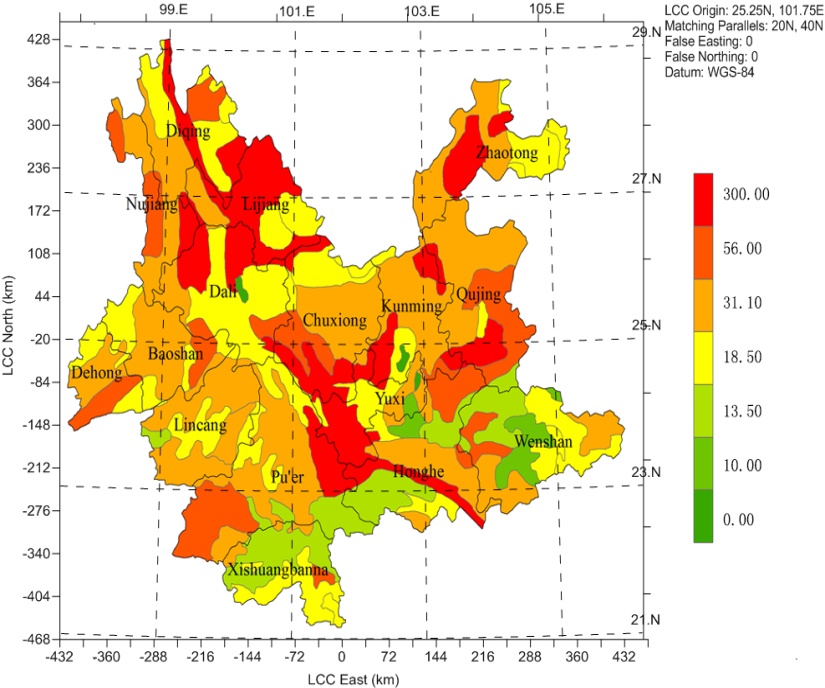


**Figure D The background of lead concentration in the soil in 1990 (shaded, mg/kg)**


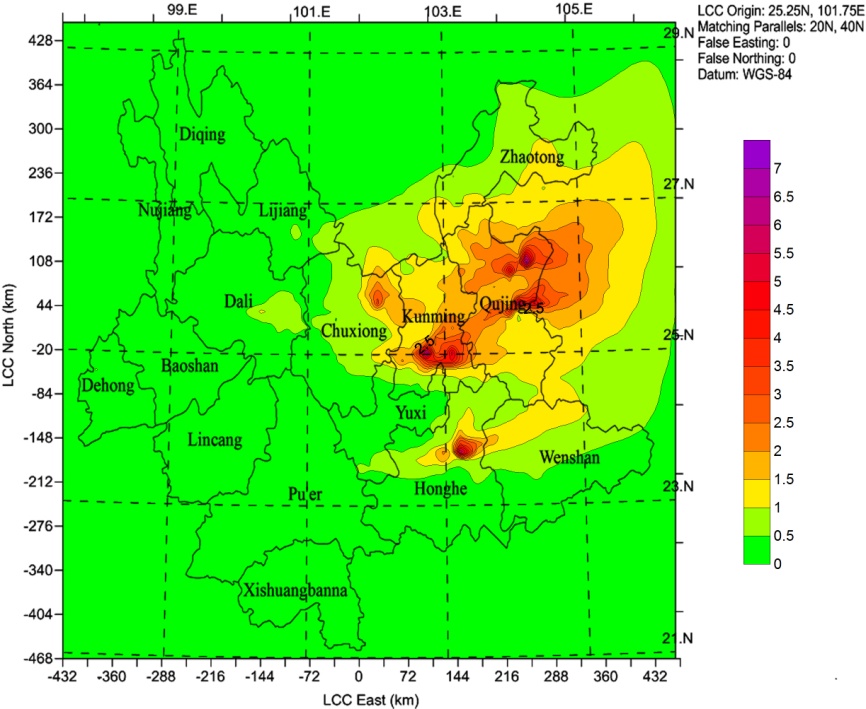


**Figure E Horizontal distributions of predicted cumulative lead concentration in topsoil from atmospheric deposition of lead (shaded, mg/kg)**


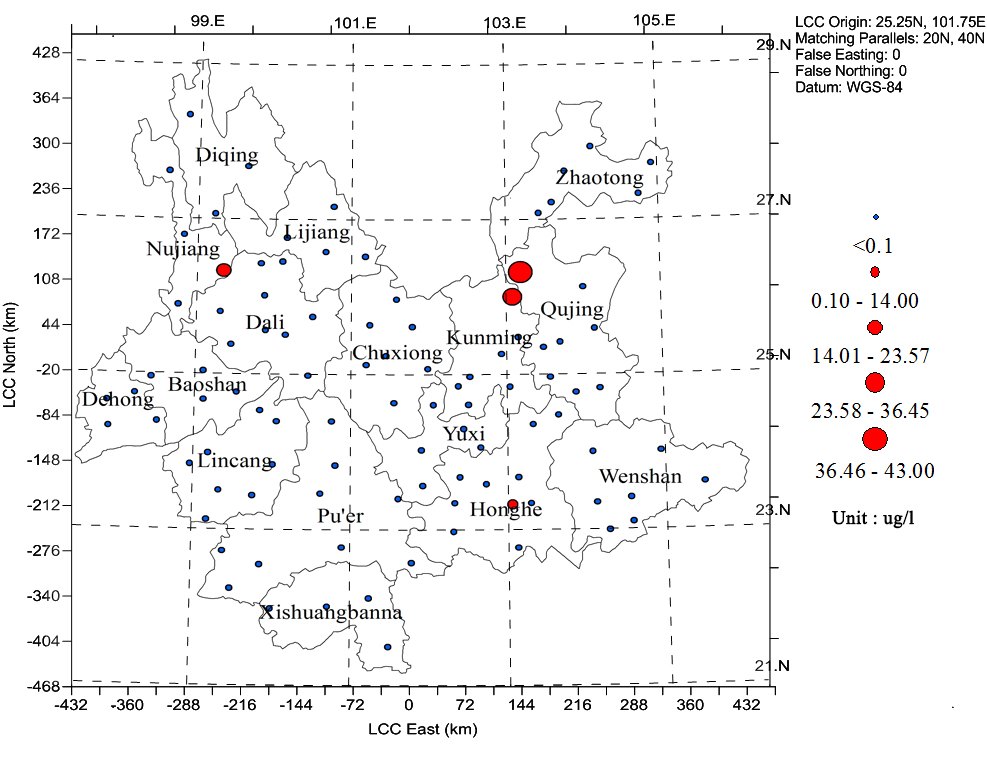


**Figure F The distribution of lead concentration in drinking water from samples**


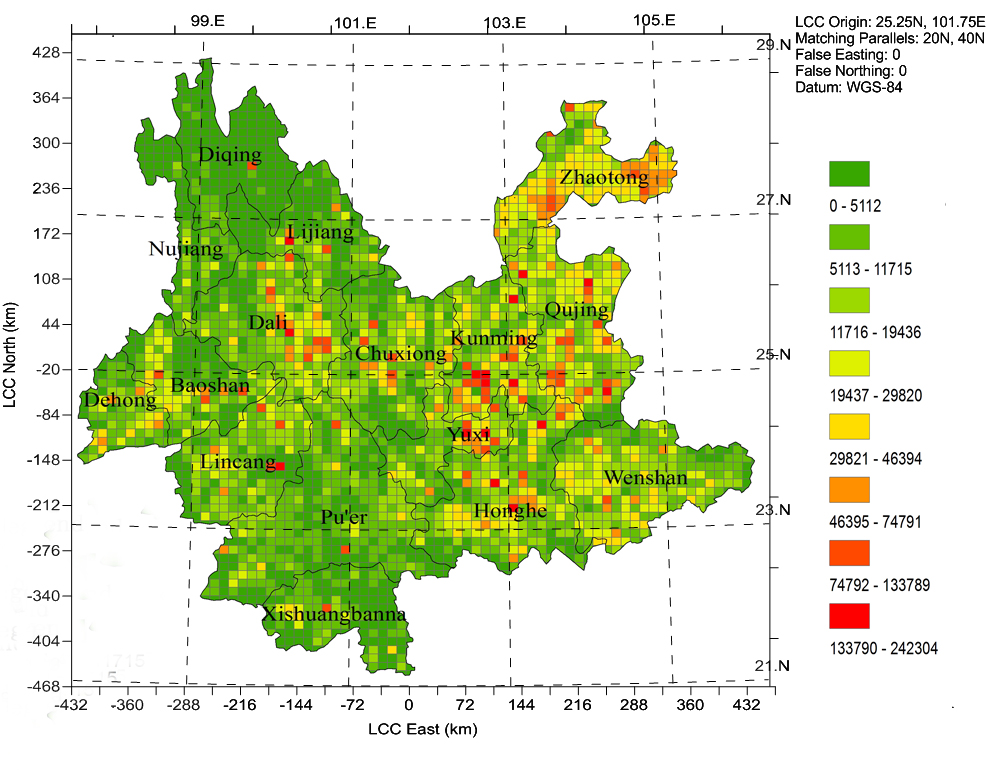


**Figure G The distribution of population in Yunnan Province in 2009**


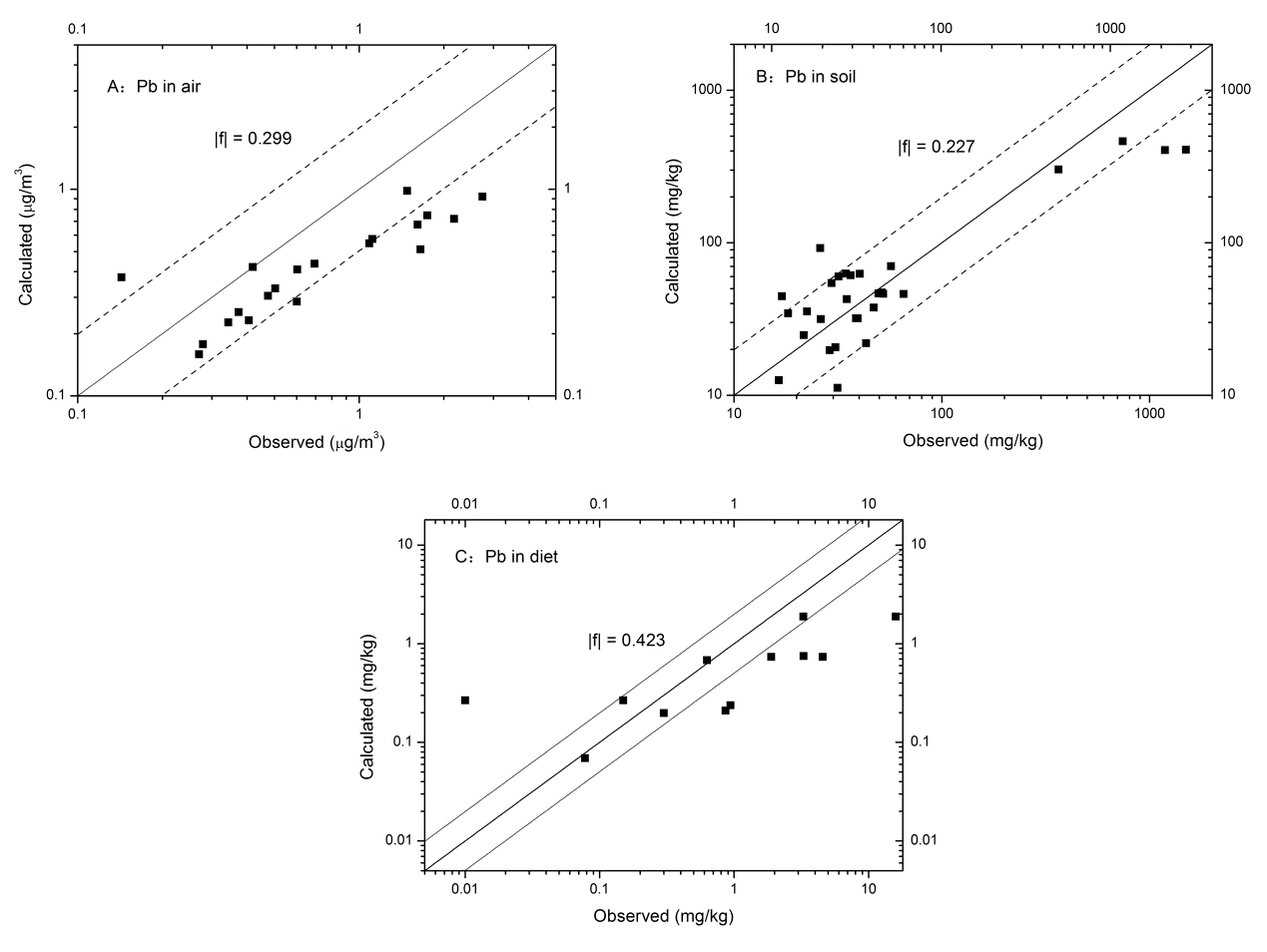


**Figure H Scatter plot of lead concentrations in Multimedia (air, soil and diet) for the model results and observations in Yunnan province.**

Note: Dots on the solid line represent a perfect fit between calculation results and observations, and dots between the two dashed lines represent error factors of less than 2. The f value is the fractional difference averaged for all pairs of the model results and observations. Generally good agreement in air, soil, and underestimation in food were demonstrated by the f value.


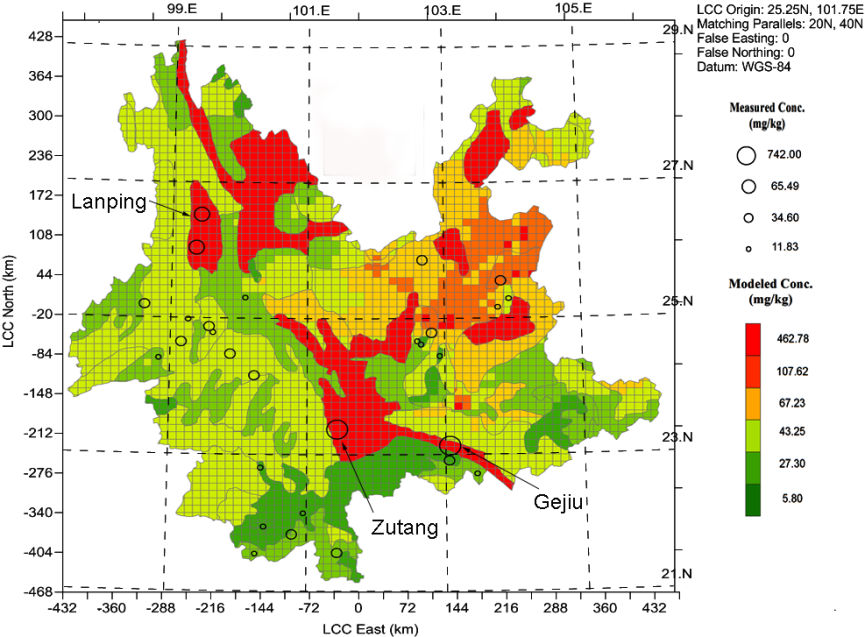


**Figure I Comparison of the modeled (white-black color contour) and measured (scaled circles) spatial distributions of lead concentration in soil in the study area.**


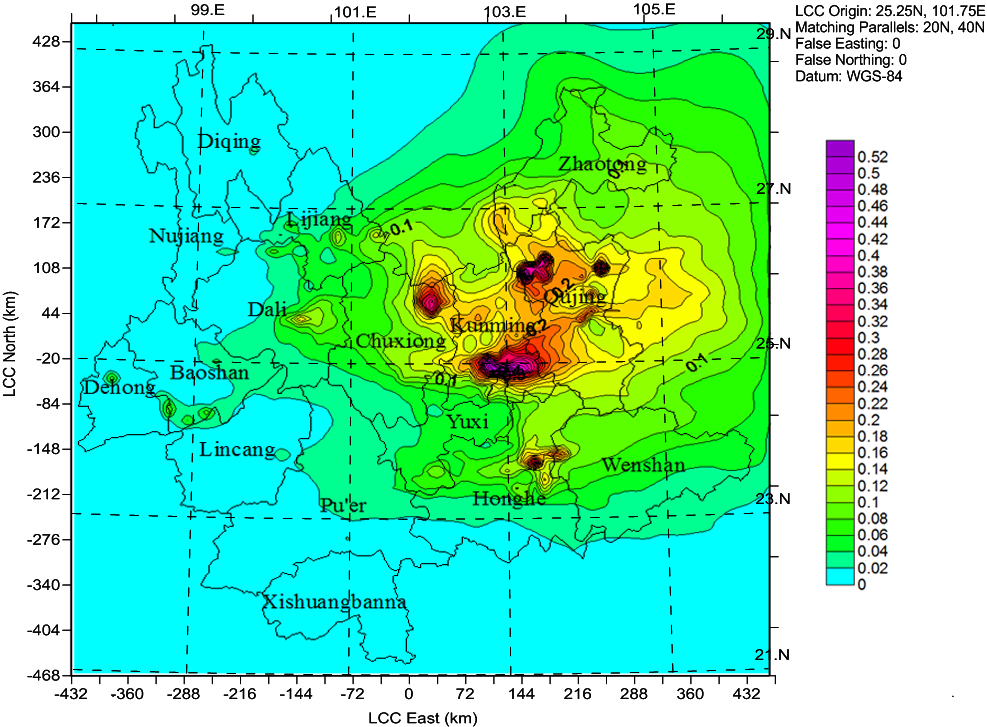


**Figure J Distribution of the modeled annual mean concentrations of lead in air in the study area, the color scale is in units of μg m^-3^**


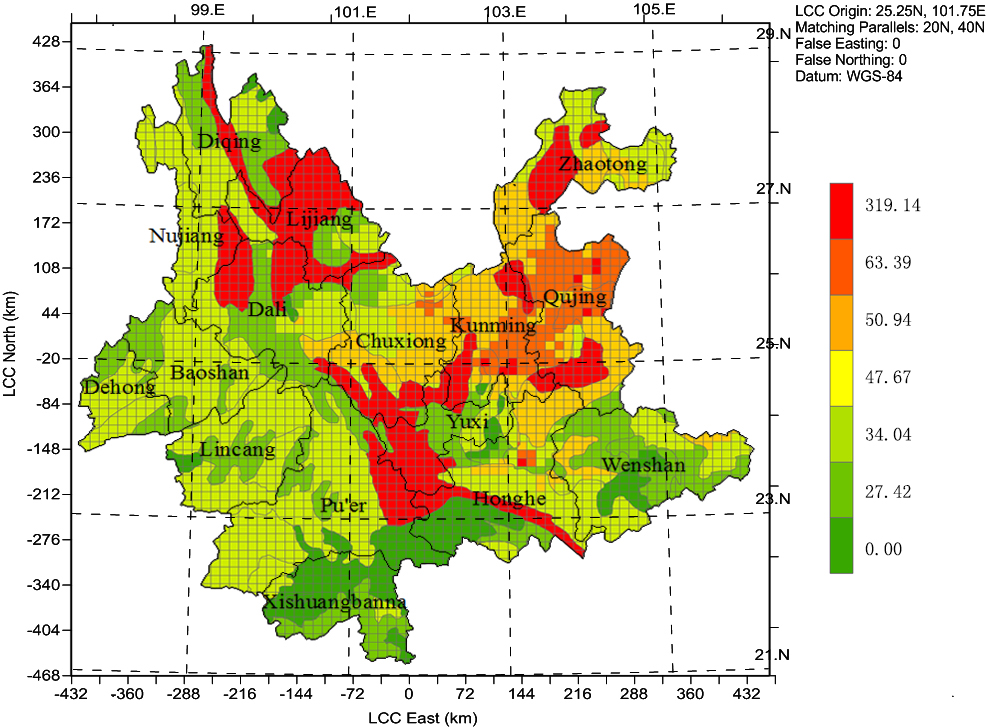


**Figure K The concentration of lead in the top soil after third years deposition**


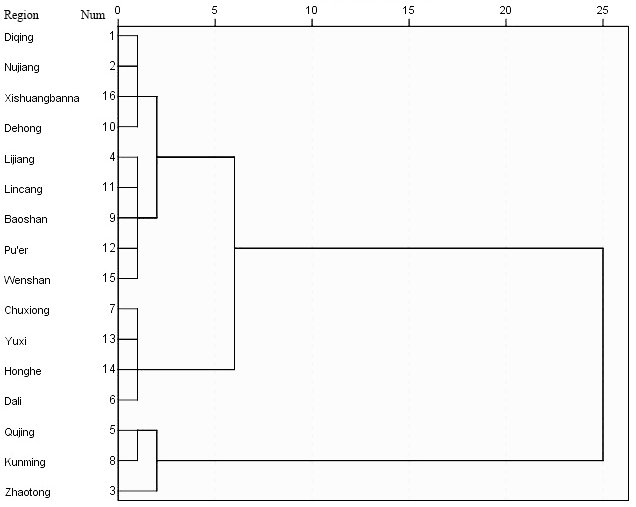


**Figure L Cluster Analysis conclusion of regional health risk level of lead pollution in Yunnan province**

**Reference**

1. Zhang Y (2006) Influencing Factor and Variation Rule of Lead Concentration in the Air in Geji. Yunnan Environmental Science (China) 25: 122-123.

2. Kasibhatla P, Chameides W, John JS (1997) A three‐dimensional global model investigation of seasonal variations in the atmospheric burden of anthropogenic sulfate aerosols. Journal of Geophysical Research: Atmospheres (1984–2012) 102: 3737-3759.

3. Wang S, Zhang J (2006) Blood lead levels in children, China. Environmental research 101: 412-418.

4. He K, Wang S, Zhang J (2009) Blood lead levels of children and its trend in China. Science of the Total Environment 407: 3986-3993.

5. Schuhmacher M, Meneses M, Xifró A, Domingo JL (2001) The use of Monte-Carlo simulation techniques for risk assessment: study of a municipal waste incinerator. Chemosphere 43: 787-799.

6. Lonati G, Zanoni F (2012) Probabilistic health risk assessment of carcinogenic emissions from a MSW gasification plant. Environment international 44: 80-91.

7. Bakoglu M, Karademir A, Durmusoglu E (2005) Evaluation of PCDD/F levels in ambient air and soils and estimation of deposition rates in Kocaeli, Turkey. Chemosphere 59: 1373-1385.

8. Lonati G, Cernuschi S, Giugliano M, Grosso M (2007) Health risk analysis of PCDD/F emissions from MSW incineration: comparison of probabilistic and deterministic approaches. Chemosphere 67: S334-S343.

9. Zhang H, Luo Y, Zhang H, Song J, Chen Y, et al. (2010) [Characterizing the plant uptake factor of As, Cd and Pb for rice and wheat cereal]. Huan jing ke xue= Huanjing kexue/[bian ji, Zhongguo ke xue yuan huan jing ke xue wei yuan hui" Huan jing ke xue" bian ji wei yuan hui] 31: 488.

10. Jia L, Wang W, Li Y, Yang L (2010) Heavy metals in soil and crops of an intensively farmed area: a case study in Yucheng City, Shandong Province, China. International journal of environmental research and public health 7: 395-412.

11. Zhuang P, McBride MB, Xia H, Li N, Li Z (2009) Health risk from heavy metals via consumption of food crops in the vicinity of Dabaoshan mine, South China. Science of the Total Environment 407: 1551-1561.

12. Pizzol M, Thomsen M, Andersen MS (2010) Long-term human exposure to lead from different media and intake pathways. Science of the Total Environment 408: 5478-5488.

13. Goodrum PE, Diamond GL, Hassett JM, Johnson DL (1996) Monte Carlo modeling of childhood lead exposure: development of a probabilistic methodology for use with the USEPA IEUBK model for lead in children. Human and Ecological Risk Assessment 2: 681-708.

14. LaGoy PK (1987) Estimated soil ingestion rates for use in risk assessment. Risk Analysis 7: 355-359.

15. Dong Z, Hu J (2011) Development of Lead Source-specific Exposure Standards Based on Aggregate Exposure Assessment: Bayesian Inversion from Biomonitoring Information to Multipathway Exposure. Environmental science & technology 46: 1144-1152.

16. YSB (2009) Yunnan statistical yearbook 2009. Beijing: China Statistics Press.

17. Liu C, Duan J, Zhenghua, Liang Z, Luo X (2011) Genetic differentiation and the characteristics of uptake and accumulation of lead among Camellia sinensis populations under different background lead concentrations of soils in Yunnan,China. Acta Ecologica Sinica (Chinese) 31: 2253-2262.

18. Shao G, Yang M, Wang L (2012) The analysis and evaluation of heavy metal pollution of vegetable soil, Baoshan City Agro-Environment & Development (Chinese) 29: 93-94.

19. SHI J, ZHANG N-m (2010) The Distributing Character of Heavy Metals and Its Pollution Estimate in Greenhouse soils of Yunnan Province. Journal of Yunnan Agricultural University (Natural Science) 6: 024.

20. Zhao X-Q, Li L-J, Yang H-H, Tan S-C (2012) The Geochemical Characteristics of Heavy Metals in Agricultural Soils of the Bijiang Watershed in Yunnan Province. Diqiu Xuebao(Acta Geoscientica Sinica) 33: 331-340.

21. Xiao-wen L (2008) Monitoring and Pollution Evaluation of Heavy Metals in the Tea Garden Soils from Main Tea Areas of Yunnan [J]. Journal of Anhui Agricultural Sciences 33: 157.

22. Xiao Q, Wang H, Zhao B, Ye Z (2011) Heavy metal pollution in crops growing in suburb of Gejiu City, Yunnan Province, China: present situation and health risk. Journal of Agro-Environment Science 30: 271-281.

23. Bai J, Xiao R, Gong A, Gao H, Huang L (2011) Assessment of heavy metal contamination of surface soils from typical paddy terrace wetlands on the Yunnan Plateau of China. Physics and Chemistry of the Earth, Parts A/B/C 36: 447-450.

24. Chen J, Zhang N, Qin L, Chen H (2004) Heavy metal pollution and pesticide residues in soils of Kunming area. Rural Eco-environment 4: 009.

25. Zhao X-Q, Yang H-H, Yi Q (2012) Characteristics and Reasons of Heavy Metal Pollution in Farmland Soils in Bijiang Watershed [J]. Journal of Anhui Agricultural Sciences 12: 215.

26. Yang S, Zhou D, Yu H, Wei R, Pan B (2013) Distribution and speciation of metals (Cu, Zn, Cd, and Pb) in agricultural and non-agricultural soils near a stream upriver from the Pearl River, China. Environmental Pollution 177: 64-70.

27. Ping D (2012) Investigations of Heavy Metals and Pesticide Contents in Soils and Vegetables inTonghai County of Yunnan [J]. Journal of Anhui Agricultural Sciences 1: 067.

28. Li Y, Schvartz C (2003) Concentration and Evaluation on pollution of Pd, Cd, Cu and Zn in vegetable farm soil and segetable of Kunming. Journal of Yunnan Agricultural University 19: 457-461.

29. Yuan Q (2010) Research on heavy metal Pollution in environment and its imPact on the major agrieultural Products in Yunnan LanPin: Dali College.
